# Supplementary material for: Identification and functional analysis of non-coding regulatory small RNA FenSr3 in Bacillus amyloliquefaciens LPB-18
Source: PeerJ. 2023 May 15;11:e15236. doi: 10.7717/peerj.15236 (PMC10194069; doi:10.7717/peerj.15236)
Supplement: Supplemental Information 4 [file peerj-11-15236-s004.zip › KO/CK-vs-T1_map/map03420.html]

KEGG PATHWAY: Nucleotide excision repair - Reference pathway


|  |  |
| --- | --- |
| **Nucleotide excision repair - Reference pathway** |  |

[
Pathway menu
| Organism menu
| Pathway entry
| Show description
| User data mapping
]

Nucleotide excision repair (NER) is a mechanism to recognize and repair bulky DNA damage caused by compounds, environmental carcinogens, and exposure to UV-light. In humans hereditary defects in the NER pathway are linked to at least three diseases: xeroderma pigmentosum (XP), Cockayne syndrome (CS), and trichothiodystrophy (TTD). The repair of damaged DNA involves at least 30 polypeptides within two different sub-pathways of NER known as transcription-coupled repair (TCR-NER) and global genome repair (GGR-NER). TCR refers to the expedited repair of lesions located in the actively transcribed strand of genes by RNA polymerase II (RNAP II). In GGR-NER the first step of damage recognition involves XPC-hHR23B complex together with XPE complex (in prokaryotes, uvrAB complex). The following steps of GGR-NER and TCR-NER are similar.

|  |  |  |
| --- | --- | --- |
| Reference pathway | 184% 150% 122% 100% 82% 67% 55% | 图片下载 |
